# Occupational exposures and genetic susceptibility to occupational exposures are related to sickness absence in the Lifelines cohort study

**DOI:** 10.1038/s41598-020-69372-6

**Published:** 2020-07-31

**Authors:** Md. Omar Faruque, Kim De Jong, Judith M. Vonk, Hans Kromhout, Roel Vermeulen, Ute Bültmann, H. Marike Boezen

**Affiliations:** 1 University of Groningen, University Medical Center Groningen, Department of Epidemiology, Hanzeplein 1, 9713 GZ Groningen, The Netherlands; 2 University of Groningen, University Medical Center Groningen, Groningen Research Institute for Asthma and COPD (GRIAC), Groningen, The Netherlands; 3 University of Utrecht, Institute for Risk Assessment Sciences (IRAS), Division of Environmental Epidemiology, Utrecht, The Netherlands; 4 University of Groningen, University Medical Center Groningen, Department of Health Sciences, Community and Occupational Medicine, Groningen, The Netherlands

**Keywords:** Health occupations, Risk factors

## Abstract

In this cross-sectional study, we investigated the association between occupational exposures and sickness absence (SA), the mediating role of respiratory symptoms, and whether genetic susceptibility to SA upon occupational exposures exists. Logistic regression was used to examine associations and structural equation modelling was used for mediation analyses. Genetic susceptibility was investigated by including interactions between occupational exposures and 11 candidate single nucleotide polymorphisms (SNPs). Biological dust, mineral dust, and pesticides exposure were associated with a lower prevalence of any SA (OR (95% CI) = 0.72 (0.58–0.89), 0.88 (0.78–0.99), and 0.70 (0.55–0.89), respectively) while gases/fumes exposure was associated with a higher prevalence of long-term SA (1.46 (1.11–1.91)). Subjects exposed to solvents and metals had a higher prevalence of any (1.14 (1.03–1.26) and 1.68 (1.26–2.24)) and long-term SA (1.26 (1.08–1.46) and 1.75 (1.15–2.67)). Chronic cough and chronic phlegm mediated the association between high gases/fumes exposure and long-term SA. Two of 11 SNPs investigated had a positive interaction with exposure on SA and one SNP negatively interacted with exposure on SA. Exposure to metals and gases/fumes showed a clear dose–response relationship with a higher prevalence of long-term SA; contrary, exposure to pesticides and biological/mineral dust showed a protective effect on any SA. Respiratory symptoms mediated the association between occupational exposures and SA. Moreover, gene-by-exposure interactions exist.

## Introduction

Sickness absence has a negative effect on production both qualitatively and quantitatively^1^. Studies from different countries and various occupational settings have shown that many factors such as socio-demographic and personal factors, physical and psychosocial working conditions as well as somatic and mental health conditions, are associated with sickness absence^2^. However, whether occupational exposure to biological dust, mineral dust, gases/fumes, pesticides, solvents, and metals is associated with sickness absence in the general working population, has yet to be elucidated.

Several studies have shown that occupational exposure to vapours, dust, gases and fumes (VGDF), organic dust, chlorinated solvents, lead, and occupational chemicals such as detergents, surfactants or pesticides, increases the prevalence of respiratory symptoms (e.g. cough, dyspnea), respiratory diseases, lung function limitation, dizziness, anxiety, abdominal pain, and skin irritation and lesions^3–5^. On inhalation, occupational exposures may impair lung function by triggering immune or inflammatory responses^6–8^. Indeed, a previous study has found that bioaerosol inhalation induced inflammation (increased neutrophils and interleukin-8 level) in the small airways, which in turn reduced lung function among organic waste collectors^9^. VGDF exposure was also strongly associated with a higher prevalence of sickness absence among workers with respiratory symptoms^10^. Therefore, it can be hypothesized that the prevalence of sickness absence is higher among people in “dirty” jobs (e.g. welding, painting, and construction) compared to people with a clean working environment, because airborne occupational exposures may affect the respiratory system. However, not all workers experience respiratory symptoms upon occupational exposure, and also the symptom severity differs between individuals^11,12^. Genetic make-up may play a role in the differential susceptibility to these exposures. Indeed, we have previously shown that specific single nucleotide polymorphisms (SNPs) in biologically plausible genes were associated with the susceptibility to occupational exposures with regard to respiratory health effects, i.e., lung function level^13,14^. For example, subjects carrying the minor allele of SNP rs17490056 had a lower FEV_1_ compared to wildtype subjects, yet only in those subjects with high biological dust exposure and not in subjects with low or no exposure^13^. These SNPs may be plausible candidates to modify the association between occupational exposures and sickness absence.

The main aim of this study was to investigate the association between occupational exposure to biological dust, mineral dust, gases/fumes, pesticides, solvents, and metals, assessed with ALOHA + job-exposure matrix (JEM)^15^, and self-reported sickness absence in active workers in a general population cohort. We further investigated whether the associations were mediated by respiratory symptoms, and we assessed whether workers with a specific genetic make-up are more susceptible to sickness absence upon occupational exposure.

## Materials and methods

### Study population

In this study, we included adults from the Lifelines cohort study and biobank^16^. At the baseline visit, between 2006 and 2013, subjects had a physical examination and completed questionnaires on occupation, health, lifestyle, environment, and psychosocial parameters. A subset (n = 13,302) of genetically unrelated Lifelines participants had genome-wide genotyping data. For this subset, we also estimated occupational exposures using a JEM. This study was approved by the Medical Ethical Commission (METC) of the University Medical Center Groningen (Reference number-2007/152). All subjects signed written informed consent. All methods were carried out in accordance with relevant guidelines and regulations for human subjects.

In the current analysis, out of 13 302 subjects, we included 10 087 ‘active workers’, defined as having a paid current job. Of those, 9,883 (98%) active workers answered the questions on sickness absence.

### Sickness absence

Sickness absence was self-reported (see ‘S1 Appendix: Supplementary Questions’). ‘Any sickness absence’ was defined as being absent from work due to illness or problems (except pregnancy) at least one day in the last year (yes/no). ‘Long-term sickness absence’ was defined as being absent from work due to illness or problems (except pregnancy) for two consecutive weeks or more in the last year (yes/no).

### Occupational exposures

Occupational exposures were estimated using the job titles as reported in the questionnaire. The self-reported jobs were coded according to the International Standard Classification of Occupations (ISCO-88)^17^. Subsequently, the ALOHA + JEM (a modified version of the ad hoc JEM for COPD called the ALOHA JEM)^18^ was used to classify occupational exposure into no, low, or high exposure categories (0/1/2) for the following occupational exposures: biological dust, mineral dust, gases/fumes, pesticides, solvents, and metals.

### Respiratory symptoms

The presence of chronic cough, chronic phlegm, and dyspnea was self-reported (see S1 Table for the exact definition).

### Candidate SNPs and genotyping

The selection of candidate SNPs was based on two genome-wide interaction studies conducted by our research group^13,14^ that identified 11 SNPs in biologically plausible genes that significantly interacted with occupational exposures on lung function, i.e. rs17490056 with biological dust, rs13278529, rs473892, and rs6751439 with mineral dust, rs159497, rs516732, and rs2888674 with gases/fumes^13^, and rs4764419, rs10459067, rs482555, and rs2145067 with pesticides^14^. Gene annotation, biological plausibility, and details on how genotyping was performed are described elsewhere^13,14^. See S2 Table for the basic information of these SNPs.

### Co-variates

Subjects’ age, sex, and body mass index (BMI) were determined during the baseline screening examination. Smoking status, monthly income, and education were taken from the baseline questionnaire. Smoking status was categorized into never, former, and current smoker. Monthly income was categorized into low, medium, high, and don’t know/don’t tell. Finally, education was categorized into low, medium, high, and unclassifiable (see ‘S3 Table’).

### Statistical methods

Chi-Square and Mann–Whitney U test were performed to investigate the univariate association of demographic characteristics, respiratory symptoms, and occupational exposures with sickness absence. To investigate the independent association between occupational exposures and sickness absence, multivariate logistic regression models with adjustment for potential confounders were used. No sickness absence was considered as reference group for both any and long-term sickness absence. Subjects with long-term sickness absence (≥ 2 weeks) were also included in the analyses on any sickness absence. In addition, we included all six airborne exposures (no vs. any exposure) in one model to assess the effect of co-exposure. A two-sided *p* value < 0.05 was considered statistically significant.

**Figure 1:**
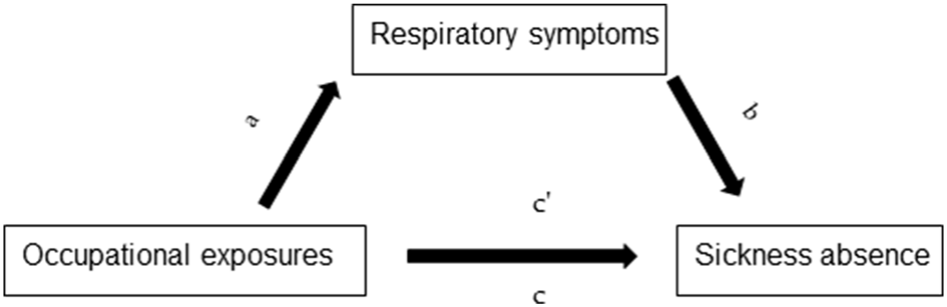
Mediation analyses pathway. Association between occupational exposures and respiratory symptoms (**a**). Association between respiratory symptoms and sickness absence (**b**). Indirect effect is a product of ab. Total effect—association between occupational exposures and sickness absence adjusted for covariates (**c**). Direct effect—association between occupational exposures and sickness absence additionally adjusted for respiratory symptoms (**c′**).

To assess whether respiratory symptoms mediate the association between occupational exposures and sickness absence, we performed structural equation modeling adjusted for covariates (Fig. 1) in MPlus software using the logit function^19^. We performed mediation analyses by respiratory symptoms for all models with a significant positive association between exposure (either high or low) and sickness absence. Significant mediation by the respiratory symptom was considered present when the *p* value of the indirect effect was < 0.05.

To assess whether the association between occupational exposures and sickness absence was dependent on genetic make-up, a multivariate logistic regression, including interactions between SNPs and occupational exposures, was used. SNPs were tested in a co-dominant model. Both SNP by low and SNP by high exposure interactions were assessed, and interaction was considered statistically significant at *p* value < 0.05. The interaction models included dummy variables for low and high exposure, for the heterozygous (HZ) and homozygous for the minor allele (HM) genotypes and their interactions i.e.

$$\begin{aligned} & {\text{Sickness}}\,{\text{absence}} = {\text{low}}\,{\text{exposure}} + {\text{high}}\,{\text{exposure}} + {\text{HZ}} + {\text{HM}} + {\text{low}}\,{\text{exposure}} \times {\text{HZ}} \\ & \;\; + {\text{low}}\,{\text{exposure}} \times {\text{HM}} + {\text{high}}\,{\text{exposure}} \times {\text{HZ}} + {\text{high}}\,{\text{exposure}} \times {\text{HM}} + {\text{covariates}} \\ \end{aligned}$$

SPSS 22 (IBM Corp. Released 2013. IBM SPSS Statistics for Windows, Version 22.0. Armonk, NY: IBM Corp) was used for the data analysis.

## Results

### Baseline characteristics

**Figure 2:**
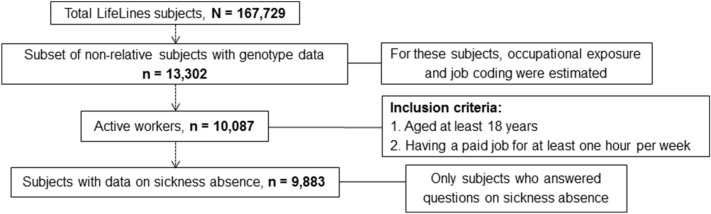
Flowchart shows selection of study subjects.

In Fig. 2, a flowchart of the subject selection is presented. In the final analyses, 204 workers were excluded because they lacked data on sickness absence. These excluded workers were slightly older, more often female, more often current smokers, and had a lower socioeconomic status compared to workers with data on sickness absence (S4 Table).

More than half of the 9,883 included subjects (53%) reported any sickness absence during the last year (Table 1). Subjects with any sickness absence were younger, more often female, had a higher BMI, were more often current smokers, had higher education but lower income, and had a higher prevalence of respiratory symptoms compared to subjects with no sickness absence. Of 5,235 subjects who reported any sickness absence, 1,230 (≈ 23%) subjects also reported long-term sickness absence. The subjects with long-term sickness absence were more often female, had a higher BMI, were more often current smokers, had lower education and lower monthly income, and had a higher prevalence of respiratory symptoms compared to subjects with no sickness absence. The distribution of the population characteristics according to the different levels of occupational exposures is given in Tables S5 and S6.

**Table 1:** Comparison of demographic factors, respiratory symptoms, and occupational exposures among subjects with no sickness absence, any sickness absence, and long-term sickness absence (≥ 2 weeks).

Demographic factors, respiratory symptoms, and occupational exposures	No sickness absence, (N = 4,648)	Any sickness absence, (5,235)	Long-term sickness absence, (1,230)
**Age** (years), Median (min–max)	46 (18–76)	45 (20–77)*	47 (20–71)
**Body mass index (BMI)** (kg/meter^2^), Median (min–max)	25 (17–53)	26 (16–52)	26 (17–51)^ϴ^
**Sex**			
Male, N (%)	2,152 (46.3)	2,269 (43.3)*	492 (40.0)^ϴ^
Female, N (%)	2,496 (53.7)	2,966 (56.7)	738 (60.0)
**Smoking status**			
Never smoker, N (%)	2064 (44.8)	2052 (39.5)*	432 (35.3)^ϴ^
Former-smoker, N (%)	1556 (33.8)	1837 (35.3)	436 (35.6)
Current smoker, N (%)	990 (21.4)	1,311 (25.2)	357 (29.1)
**Education**			
Low, N (%)	767 (16.5)	782 (15.0)*	271 (22.1)^ϴ^
Medium, N (%)	2,521 (54.3)	2,794 (53.4)	691 (56.3)
High, N (%)	1,341 (28.9)	1647 (31.5)	265 (21.6)
Unclassifiable, N (%)	13 (0.3)	6 (0.1)	1 (0.1)
**Monthly income**			
Low income, N (%)	390 (8.4)	597 (11.4)*	178 (14.5)^ϴ^
Medium income, N (%)	1,283 (27.7)	1579 (30.3)	403 (32.9)
High income, N (%)	2,197 (47.5)	2,478 (47.5)	479 (39.1)
Don’t know/don’t tell, N (%)	757 (16.4)	565 (10.8)	164 (13.4)
**Chronic cough**			
No, N (%)	4,322 (93.9)	4,699 (90.9)*	1,091 (89.6)^ϴ^
Yes, N (%)	283 (6.1)	473 (9.1)	126 (10.4)
**Chronic phlegm**			
No, N (%)	4,389 (95.0)	4,801 (92.3)*	1,110 (91.1)^ϴ^
Yes, N (%)	230 (5.0)	400 (7.7)	109 (8.9)
**Dyspnea**			
No, N (%)	3,502 (87.8)	3,525 (82.3)*	733 (76.9)^ϴ^
Yes, N (%)	488 (12.2)	756 (17.7)	220 (23.1)
**Biological dust**			
No exposure, N (%)	3,188 (68.6)	3,574 (68.3)*	782 (63.6)^ϴ^
Low exposure, N (%)	1,233 (26.5)	1,498 (28.6)	397 (32.3)
High exposure, N (%)	227 (4.9)	163 (3.1)	51 (4.1)
**Mineral dust**			
No exposure, N (%)	3,624 (78.0)	4,198 (80.2)*	922 (75.0)^ϴ^
Low exposure, N (%)	801 (17.2)	790 (15.1)	230 (18.7)
High exposure, N (%)	223 (4.8)	247 (4.7)	78 (6.3)
**Gases/fumes**			
No exposure, N (%)	2,672 (57.5)	3,061 (58.5)	613 (49.8)^ϴ^
Low exposure, N (%)	1696 (36.5)	1853 (35.4)	516 (42.0)
High exposure, N (%)	280 (6.0)	321 (6.1)	101 (8.2)
**Pesticides**			
No exposure, N (%)	4,412 (94.9)	5,077 (97.0)*	1,180 (95.9)
Low exposure, N (%)	184 (4.0)	124 (2.4)	37 (3.0)
High exposure, N (%)	52 (1.1)	34 (0.6)	13 (1.1)
**Solvents**			
No exposure, N (%)	3,519 (75.7)	3,819 (73.0)*	871 (70.8)^ϴ^
Low exposure, N (%)	972 (20.9)	1,232 (23.5)	318 (25.9)
High exposure, N (%)	157 (3.4)	184 (3.5)	41 (3.3)
**Metals**			
No exposure, N (%)	4,335 (93.3)	4,831 (92.3)*	1,114 (90.6)^ϴ^
Low exposure, N (%)	228 (4.9)	263 (5.0)	81 (6.6)
High exposure, N (%)	85 (1.8)	141 (2.7)	35 (2.8)

Subjects with long-term sickness absence were also included in the analyses on any sickness absence.

*Statistically significant at two-sided *p* value < 0.05 between no sickness absence and any sickness absence.

^ϴ^Statistically significant at two-sided *p* value < 0.05 between no sickness absence and long-term sickness absence.

### Occupational exposures and sickness absence

Table 1 shows that subjects reporting any sickness absence during the last year were somewhat more often exposed to solvents and metals, while they had a lower prevalence of high exposure to biological dust, mineral dust, and pesticides compared to subjects reporting no sickness absence. Subjects reporting long-term sickness absence were (considerably) more often exposed to biological dust, mineral dust, gases/fumes, solvents, and metals compared to subjects reporting no sickness absence. The correlation among different occupational exposures is given in S1 Fig.

**Figure 3:**
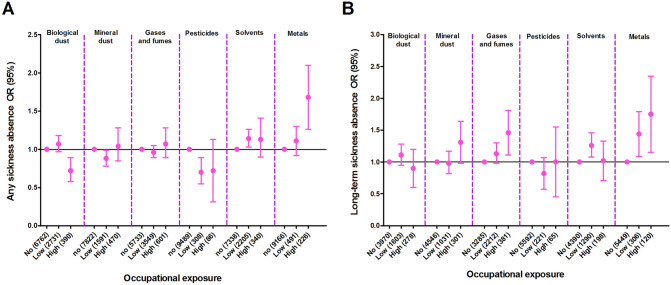
Associations between occupational exposures and sickness absence. Sickness absence presented as (**A**) Any sickness absence—subjects with long-term sickness absence were also included in the analyses on any sickness absence. (**B**) Long-term sickness absence (≥ 2 weeks). The multivariate logistic regression model was adjusted for age, sex, BMI, education, smoking status, and monthly income.

After adjustment for covariates, subjects with high exposure to biological dust and low exposure to mineral dust or pesticides had a lower prevalence of any sickness absence compared to subjects without these exposures (Fig. 3). No significant associations were found between these exposures and long-term sickness absence. Subjects with high exposure to gases/fumes reported long-term sickness absence more often, but not any sickness absence, compared to subjects not exposed to gases/fumes. Low exposure to solvents was associated with a higher prevalence of both any and long-term sickness absence. High exposure to metals was associated with a higher prevalence of any sickness absence whereas both exposure to low and high metals were associated with a higher prevalence of long-term sickness absence in a dose dependent way (see S7 Table).

In the co-exposure analyses, the results are consistent with the main results in terms of direction of the coefficients. The co-exposure results showed that exposure to gases/fumes and pesticides was associated with a lower prevalence of any sickness absence while metals exposure was associated with a higher prevalence of both any and long-term sickness absence (Table S8).

### Mediation analyses

The significant association between high exposure to gases/fumes and long-term sickness absence was mediated by chronic cough and chronic phlegm (Table 2). The association between airborne exposures and respiratory symptoms and the association between respiratory symptoms and sickness absence are given in Table S9 and S10, respectively. The association between high solvents exposure and any sickness absence was mediated by chronic phlegm; however, the association between high solvents exposure and any sickness absence was not significant. The associations between low and high metals exposure and sickness absence were not mediated by respiratory symptoms.

**Table 2:** Mediation analysis of respiratory symptoms in the association between occupational exposures and sickness absence.

Occupational exposures	Any sickness absence						Long-term sickness absence					
	Total effect		Direct effect		Indirect effect		Total effect		Direct effect		Total indirect effect	
	OR (95% CI)	*p*	OR (95% CI)	*p*	OR (95% CI)	*p*	OR (95% CI)	*p*	OR (95% CI)	*p*	OR (95% CI)	*p*
**Chronic cough**												
**Gases/fumes**												
Low exposure							1.13 (0.96–1.29)	0.128	1.12 (0.96–1.28)	0.137	1.00 (1.00–1.01)	0.233
High exposure							**1.45 (1.06–1.85)**	**0.028**	**1.44 (1.05–1.83)**	**0.031**	**1.01 (1.00–1.02)**	**0.048**
**Solvents**												
Low exposure	**1.14 (1.03–1.25)**	**0.008**	**1.14 (1.03–1.25)**	**0.009**	1.00 (1.00–1.01)	0.389	**1.26 (1.06–1.48)**	**0.023**	**1.25 (1.06–1.44)**	**0.024**	1.00 (0.98–1.01)	0.407
High exposure	1.12 (0.87–1.37)	0.319	1.12 (0.86–1.37)	0.336	1.00 (0.99–1.02)	0.504	1.02 (0.65–1.39)	0.911	1.02 (0.65–1.38)	0.929	1.00 (0.99–1.02)	0.514
**Metals**												
Low exposure	1.11 (0.90–1.32)	0.284	1.11 (0.90–1.32)	0.291	1.00 (0.99–1.01)	0.758	**1.44 (1.03–1.85)**	**0.042**	**1.44 (1.03–1.84)**	**0.042**	1.00 (0.99–1.01)	0.754
High exposure	**1.70 (1.21–2.18)**	**0.000**	**1.71 (1.22–2.20)**	**0.000**	0.99 (0.98–1.01)	0.385	**1.76 (1.02–2.50)**	**0.048**	**1.77 (1.02–2.52)**	**0.046**	0.99 (0.98–1.01)	0.401
**Chronic phlegm**												
**Gases/fumes**												
Low exposure							1.13 (0.97–1.29)	0.122	1.13 (0.96–1.29)	0.129	1.00 (0.98–1.01)	0.350
High exposure							**1.47 (1.07–1.87)**	**0.025**	**1.44 (1.05–1.83)**	**0.031**	**1.02 (1.01–1.04)**	**0.022**
**Solvents**												
Low exposure	**1.14 (1.03–1.25)**	**0.008**	**1.14 (1.03–1.25)**	**0.009**	1.00 (0.99–1.01)	0.803	**1.26 (1.07–1.45)**	**0.023**	**1.26 (1.07–1.49)**	**0.023**	1.00 (0.99–1.01)	0.806
High exposure	1.12 (0.87–1.38)	0.315	1.11 (0.86–1.36)	0.375	**1.01 (1.00–1.03)**	**0.049**	1.03 (0.66–1.41)	0.867	1.01 (0.65–1.38)	0.935	1.02 (1.00–1.03)	0.096
**Metals**												
Low exposure	1.11 (0.90–1.33	0.273	1.11 (0.89–1.32)	0.301	1.01 (1.00–1.02)	0.240	**1.45 (1.04–1.85)**	**0.040**	**1.44 (1.03–1.84)**	**0.043**	1.01 (0.99–1.02)	0.268
High exposure	**1.70 (1.21–2.19)**	**0.000**	**1.67 (1.19–2.15)**	**0.000**	1.01 (1.00–1.03)	0.078	**1.78 (1.03–2.53)**	**0.046**	1.75 (1.01–2.49)	0.050	1.02 (1.00–1.04)	0.118
**Dyspnea**												
**Gases/fumes**												
Low exposure							1.13 (0.97–1.30)	0.113	1.13 (0.97–1.29)	0.117	1.00 (0.99–1.01)	0.687
High exposure							**1.45 (1.06–1.85)**	**0.028**	**1.44 (.105–1.83)**	**0.030**	1.01 (0.99–1.03)	0.454
**Solvents**												
Low exposure	**1.14 (1.03–1.25)**	**0.009**	**1.14 (1.03–1.26)**	**0.007**	1.00 (0.99–1.00)	0.440	**1.26 (1.07–1.46)**	**0.021**	**1.27 (1.08–1.46)**	**0.020**	1.00 (0.99–1.01)	0.439
High exposure	1.12 (0.87–1.37)	0.321	1.12 (0.87–1.37)	0.324	1.00 (0.98–1.02)	0.920	1.03 (0.65–1.40)	0.887	.Q03 (0.65–1.40)	0.892	1.00 (0.98–1.02)	0.923
**Metals**												
Low exposure	1.11 (0.90–1.32)	0.280	1.11 (0.89–1.32)	0.296	1.00 (0.99–1.02)	0.587	**1.44 (1.03–1.85)**	**0.042**	**1.43 (1.03–1.83)**	**0.044**	1.01 (0.99–1.02)	0.549
High exposure	**1.70 (1.21–2.19)**	**0.000**	**1.68 (1.20–2.16)**	**0.000**	1.01 (0.99–1.03)	0.157	**1.77 (1.02–2.52)**	**0.047**	1.73 (1.00–2.47)	0.053	1.02 (0.99–1.05)	0.187

*OR* odds ratio, *CI* confidence interval. Bold p < 0.05

### Gene-by-exposure interactions on sickness absence

**Figure 4:**
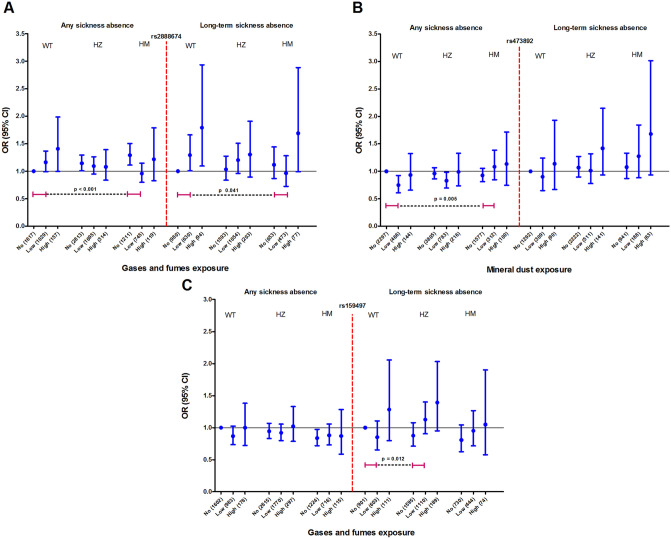
Associations between SNPs and sickness absence in subjects with no, low, and high gases and fumes and mineral dust exposure. In both any and long-term sickness absence, no exposure was considered as reference group. SNPs presented are (**A**) rs2888674, (**B**) rs473892, and (**C**) rs159497. Interactions analysis adjusted for age, sex, BMI, education, smoking status, and monthly income. WT = wild type; HZ = heterozygous; HM = Homozygous minor.

Out of the 11 candidate SNPs, three SNPs had a significant interaction with occupational exposures on sickness absence (see Fig. 4 and Tables S11–S14). Two of the SNPs (rs473892 and rs159497) had a positive interaction with exposure to mineral dust and gases/fumes, respectively, on sickness absence. This finding implies that subjects carrying one (for rs159497) or two (for rs473892) minor alleles reported a higher prevalence of long-term or any sickness absence upon the specific occupational exposure compared to subjects carrying two major alleles (Fig. 4B,C). One SNP (rs2888674) negatively interacted with exposure to gases/fumes on both any and long-term sickness absence (Fig. 4A).

## Discussion

In this large cross-sectional study, we investigated the association between occupational exposure to biological dust, mineral dust, gases/fumes, pesticides, solvents, or metals and sickness absence. We investigated whether the associations were mediated by respiratory symptoms. In addition, we explored whether subjects with a specific genotype were more susceptible to the effects of occupational exposure on sickness absence.

The results showed that subjects with high exposure to biological dust or low exposure to mineral dust and especially to pesticides significantly less often reported any sickness absence. Subjects with high exposure to gases/fumes reported long-term sickness absence significantly more often, and subjects with low exposure to solvents and high exposure to metals reported a significantly higher prevalence of both any and long-term sickness absence. For exposure to metals, long-term sickness absence increased with the intensity of exposure. The results further showed that chronic cough and chronic phlegm significantly mediate the association between high exposure to gases/fumes and long-term sickness absence.

The main route of exposure to dust and fumes is through inhalation, and this specifically affects the respiratory system. Dust and fumes exposure is a strong predictor of respiratory symptoms^3^. Also our previous study found that high exposure to dust and gases/fumes was associated with airway obstruction^4^. In addition, another study showed that any exposure to dust and fumes is strongly associated with sickness absence in subjects with respiratory symptoms^10^. Our results showed indeed positive associations between occupational exposure to gases/fumes and sickness absence, especially with long-term sickness absence and this association (partly) runs via respiratory symptoms. However, for both biological dust (high exposure) and mineral dust (low exposure), we found a lower prevalence of any sickness absence in the exposed subjects. This lower prevalence of sickness absence is also seen in subjects with low pesticide exposure. A recent meta-analysis showed negative effects of exposure to biological and mineral dust on lung function level^20^ and a recent review showed that pesticides exposure is associated with a higher incidence of chronic diseases^21^. One explanation for our unexpected findings could be that a significant proportion of subjects that were exposed to pesticides, high biological dust, and low mineral dust were self-employed workers (among those with pesticide exposure ~ 50%, high biological dust exposure ~ 40% and low mineral dust exposure ~ 10%). Farmers had a very low prevalence of any sickness absence, i.e., 22% (sickness absence prevalence in the total study sample was 53%), which may be the result of being self-employed. Previous studies showed that self-employed workers tend to have a lower prevalence of sickness absence compared to employed workers^22,23^. Possible reasons for this may be lack of compensation, high time demands, or difficulties in finding a replacer^23,24^.

An alternative explanation for our unexpected findings could be the ‘healthy worker effect’^25,26^. This implies that workers’ existing respiratory disease or symptoms could be worsened or exacerbated upon exposure to biological dust, mineral dust, or pesticides. In addition, some workers might be sensitive (in other word allergic) to these airborne occupational exposures. Thus, due to the unfavorable working environment, these workers did not take up a job with these types of exposure, or switched to a job with less occupational exposure. As a result, only the workers who did not experience negative health effects from these exposures stayed in their job.

Occupational exposure to solvents or metals was associated with a higher prevalence of sickness absence in an exposure intensity depending way. Previous studies showed that occupational exposure to solvents and metals was associated with a broad spectrum of diseases, such as pulmonary diseases, obstructive sleep apnea, brain diseases, and kidney diseases^27–29^. Given these broad ranges of health consequences of exposure to solvents and metals, it is not surprising that we found a higher prevalence of sickness absence in exposed subjects.

In addition, the co-exposure analyses showed that gases/fumes and pesticides are protective of any sickness absence while metals exposure increases the risk of both any and long-term sickness absence. These findings suggest that gases/fumes, pesticides, and metals could act through a complex biological mechanism and might follow a different biological pathway. More studies are warranted to disentangle the biological pathways through which airborne exposures act. In this study, airborne occupational exposures were not associated with respiratory symptoms. However, previous studies found a positive association between airborne exposures and respiratory symptoms^3,30,31^. Again, the healthy worker effect (discussed above) could explain our non-significant results. Indeed, a previous study showed that workers with chronic bronchitis had a 23% higher chance of quitting airborne exposure-related jobs than subjects with no such symptoms^32^.

However, an assumption-free mediation analysis (structural equation model) showed that chronic cough and chronic phlegm mediate the association between high gases/fumes exposure and long-term sickness absence. This means that high gases/fumes exposure is a risk factor for chronic cough and chronic phlegm, which in turn lead to sickness absence, especially long-term sickness absence.

The mediation effects of these symptoms only partly explain the association between exposure and sickness absence. This indicates that other factors could mediate the association between occupational exposures and sickness absence. Indeed, studies found that chronic diseases and mental disorders are strong predictors of sickness absence^2,33^. Future studies should consider these factors as potential mediators in the association between occupational exposures and sickness absence.

In the current study, we investigated whether our previously identified SNPs modify the association between occupational exposures and sickness absence. Earlier, we observed effect modification by SNPs on the association between occupational exposures and lung function^13,14^ suggesting that these genetic variants make subjects more susceptible to the health effects of occupational exposures. Previous studies showed that a lower level of forced expiratory volume in 1 s (FEV_1_)/forced vital capacity (FVC) and FVC are associated with a higher prevalence of sickness absence^34,35^. We assume that workers with limited airflow capacity might struggle to cope with the strenuous workload, and therefore, they might have repeated sickness absence periods. Thus, we expected effect modification by these identified biologically plausible SNPs on the association between occupational exposure and sickness absence. Indeed, we found several SNPs that interacted with mineral dust and gases/fumes exposure on sickness absence.

Subjects homozygous for the minor allele of rs2888674 had a lower prevalence of sickness absence upon gases/fumes exposure compared to subjects homozygous for the major allele. The minor allele of rs2888674 may thus be protective against the effects of gases/fumes exposure. In our previous study, we observed a protective effect of the rs2888674 minor allele on FEV_1_ level upon gases/fumes exposure^13^. The minor allele of rs2888674 is associated with a higher TMEM176A expression compared to the major allele^13^. A higher expression of TMEM176A attenuates co-stimulatory molecules expression and thereby, weakens inflammatory response^36^. Thus, we hypothesize that the protective effect against occupational exposure of the minor allele of rs2888674 (i.e., less sickness absence and less affected lung function level) may be the result of this lower inflammatory response to environmental triggers.

Subjects who were exposed to mineral dust and homozygous for the minor allele of rs473892 reported a higher prevalence of any sickness absence compared to exposed subjects who were homozygous for the major allele. In our previous study, rs473892 showed the same protective effect against exposure as the TMEM176A SNP described in the previous paragraph^13^. This implies that the result of the current study on sickness absence is contradicting our previous results on lung function. SNP rs473892 is located near the oligodendrocyte transcription factor 3 (OLIG3) gene, and the biological function of OLIG3 is largely unknown, more research is required to explain this finding.

Subjects heterozygous for rs159497 had a higher prevalence of long-term sickness absence upon gases/fumes exposure compared to exposed subjects homozygous for the major allele. Rs159497 is located near the phosphodiesterase-4D (PDE4D) gene, and the minor allele is associated with a higher PDE4D-expression^13^. The PDE4D-enzyme has a degrading and inactivating role on cyclic adenosine monophosphate (cAMP)^37^. cAMP attenuates immune and inflammatory responses and leads to airway smooth muscle relaxation and bronchodilation^38^. Therefore, a higher PDE4D-level may increase inflammation, and subsequently make the subject vulnerable to the harmful effects of environmental substances. Our observation that minor allele carriers exposed to gases/fumes had a higher prevalence of sickness absence is in line with this.

We did not find any significant gene-by-biological dust or gene-by-pesticides interactions on sickness absence.

### Strengths and limitations

To our knowledge, this is the first study that investigated the association between several (airborne) occupational exposures (i.e., biological dust, mineral dust, gases/fumes, pesticides, solvents, and metals) and sickness absence in the general working population. We used information from almost 10,000 extensively characterized Lifelines subjects. In addition, we investigated whether subjects with a specific genetic make-up are more susceptible to sickness absence upon occupational exposures. In developed countries such as The Netherlands, strict occupational safety and health guidelines have been developed to protect workers. Despite this, we still found a strong association between airborne occupational exposure and sickness absence. Hence, it could be questioned whether, in practice, workers fully comply with the provided preventive measures.

The JEM is an easy-to-use tool for assessing occupational exposure with several advantages. The JEM converts coded occupational titles into estimated exposures, which is advantageous in many instances when it is difficult to measure exposure at the individual level^39^. In the self-reported approach, workers often struggle to estimate exposure level when an agent is not seen or smelled^40^, and difficulty in recalling the correct exposure duration influences the validity and reliability of the report^41^. A JEM estimates occupational exposure independent of workers' perception of exposure, and thus eliminates the chance of differential misclassification or recall bias^40^. However, a JEM may result in non-differential misclassification^42^ and thereby dilutes the effect estimates towards null or insignificant values^43^. A disadvantage is that a JEM does not assess exposure at the individual chemical or biological agent level. Furthermore, this study is cross-sectional in design, so it does not infer any causality, nor does it take lifetime cumulative exposure into account. Finally, we adjusted for well-known covariates (also covariates that are available in the Lifelines cohort study) to overcome confounding effects. We did not adjust for other potential confounders such as stress, physical workload, or type of employment contract. So we cannot rule out the effect of these unmeasured confounders in our analysis.

## Conclusions

In conclusion, high exposure to gases/fumes, low exposure to solvents, and metals exposure are associated with a higher prevalence of sickness absence and especially with long-term sickness absence. Chronic cough and chronic phlegm mediate the association between high gases/fumes exposure and long-term sickness absence. Although many preventive measures are applied to control occupational exposure levels, still an association with sickness absence exists. Studying gene-by-occupational exposure interactions may help to understand underlying cellular and molecular pathways. Future research should focus on the causal association between the identified genes and health effects. A thorough understanding of the gene-by-exposure effect on health will enable us to identify susceptible subjects and set health-based and personalized recommended exposure limits for all exposed workers.

## Supplementary information

Supplementary Information.

## Data Availability

Registration is required to obtain data from the Lifelines cohort study. It is not permitted to deposit the Lifelines data in an open data repository. To obtain data, used in the current study, interested researchers should contact the Lifelines cohort study (www.lifelines.nl).
