# Supplementary material for: Occupational exposures and genetic susceptibility to occupational exposures are related to sickness absence in the Lifelines cohort study
Source: Sci Rep. 2020 Jul 31;10:12963. doi: 10.1038/s41598-020-69372-6 (PMC7395760; doi:10.1038/s41598-020-69372-6)
Supplement: Supplementary file 1 — Supplementary Information. [file 41598_2020_69372_MOESM1_ESM.pdf]

Occupational exposures and genetic susceptibility to occupational exposures are related to sickness absence in the Lifelines cohort study

Md Omar Faruque, Kim De Jong, Judith M Vonk, Hans Kromhout, Roel Vermeulen,

Ute Bültmann, H Marike Boezen

Supplementary information

S1 Appendix: Supplementary Questions

1. In the last year. how many days/weeks did you not work due to illness or problems (excluding pregnancy)? Answer: number of days or weeks.
2. In the last year. did you not go to work for one or more periods of at least two consecutive weeks because of illness or problems (excluding pregnancy)? Answer categories: Yes/No.

S1 Table: Definition of the respiratory symptoms used in the main analyses.

Respiratory symptoms	Definition
**Chronic cough**	Usual coughing first thing in the morning, or during the day, or at night in winter for at least three months in a year.
**Chronic phlegm**	Usual bringing up any phlegm first thing in the morning, or during the day, or at night in winter for at least three months in a year.
**Dyspnea**	Having shortness of breath when hurrying on level ground, or walking up a slight hill, or stairs at a normal pace.

S2 Table: Basic information of SNPs investigated in the study

SNPs	Chromosome	Effect allele	Reference allele	Effect allele frequency	Annotation
**rs17490056**	13	T	C	0.50	PCDH9 (150 Kb 39)
**rs13278529**	8	G	T	0.15	ZMAT4 (55 Kb 39)
**rs473892**	6	T	C	0.46	OLIG3 (136 Kb 59)
**rs6751439**	2	A	G	0.13	GALNT13 (intronic)
**rs159497**	5	C	T	0.46	PDE4D (57 Kb 39)
**rs516732**	5	C	T	0.47	ODZ2 (1.6 Mb 59)
**rs2888674**	7	A	G	0.46	TMEM176A (8.7 Kb 39)
**rs4764419**	12	G	T	0.46	PLCZ1 (intronic)
**rs10459067**	12	T	C	0.43	PLCZ1 (intronic)
**rs482555**	12	C	T	0.25	NOS1 (intronic)
**rs2145067**	6	T	C	0.17	MANEA (769 kb 59)

S3 Table: Co-variates adjusted in the regression analyses.

Covariates	Operational definition
**Age**	Subjects' age in years
**Body Mass Index (BMI)**	Subjects' BMI calculated as weight in kilogram/ (height in meter)^2
**Sex**	**Gender of the study subjects**
Male	
Female	
**Smoking status**	**Smoking status of the subjects**
Never smoker	Never smoked or smoked for < 1 year
Former-smoker	Smoked for ≥ 1 year and stopped smoking for ≥ 1 month
Current smoker	Current smoker or stopped smoking < 1 month
**Education**	**Highest level of completed education**
Low	No training. primary education. or lower or pre-vocational education
Medium	General secondary education. secondary vocational or professional guiding. or pre-university education
High	higher professional or university degree
Unclassifiable	Subjects with other than above-mentioned education.
**Monthly income**	**Subjects monthly income in euros**
Low income	Monthly income ≤ €1500
Medium income	Monthly income between €1500 and €2500
High income	Monthly income ≥ €2500
Don't know/Don't tell	I don't know/ I don't want to say

S4 Table: Comparison of demographic characteristics between workers with and without data on sickness absence.

Demographic factors	Workers with no data on sickness absence. 204 (2%)	Workers with data on sickness absence. 9883 (98%)	p-value
**Age** (years) Median (min-max)	48 (22-73)	46 (18-77)	**<0.001**
**Body Mass Index (BMI)** (kg/meter^2) Median (min-max)	26 (17-45)	26 (16-53)	0.277
**Sex**			
Female. N (%)	138 (67.6)	5462 (55.3)	**<0.001**
**Smoking status**			
Never smoker, N (%)	72 (35.6)	4116 (42.0)	**0.030**
Former-smoker, N (%)	67 (33.2)	3393 (34.6)	
Current smoker, N (%)	63 (31.2)	2301 (23.5)	
**Education**			
Low, N (%)	66 (33.0)	1549 (15.7)	**<0.001**
Medium, N (%)	105 (52.5)	5315 (53.8)	
High, N (%)	29 (14.5)	2988 (30.3)	
Unclassifiable, N (%)	0 (0.0)	19 (0.2)	
**Monthly Income**			
Low income, N (%)	38 (19.3)	987 (10.0)	**<0.001**
Medium income, N (%)	63 (32.0)	2862 (29.1)	
High income, N (%)	45 (22.8)	4675 (47.5)	
Don’t know/Don’t tell, N (%)	51 (25.9)	1322 (13.4)	

S5 Table: Distribution of the population characteristics according to the different levels of occupational exposures (biological dust, mineral dust, and gases/fumes).

Population characteristics	Biological dust			Mineral dust			Gases/fumes		
	No	Low	High	No	Low	High	No	Low	High
**Age, median (min-max)**	46 (18-77)	46 (20-75)	45 (22-71)	46 (18-77)	46 (21-75)	45 (20-73)	46 (18-77)	46 (20-75)	45 (23-70)
**BMI, median (min-max)**	26 (16-53)	26 (17-52)	26 (18-49)	25 (17-53)	26 (16-52)	27 (17-48)	25 (17-53)	26 (17-52)	26 (16-47)
**Sex**									
Male, N (%)	3447 (50.0)	715 (25.6)	325 (80.6)	3114 (39.1)	933 (57.1)	440 (91.1)	2491 (42.7)	1441 (39.6)	555 (90.7)
Female, N(%)	3443 (50.0)	2079 (74.4)	78 (19.4)	4856 (60.9)	701 (42.9)	43 (8.9)	3347 (57.3)	2196 (60.4)	57 (9.3)
**Smoking status**									
Never smoker, N (%)	2912 (42.5)	1085 (39.2)	191 (48.0)	3396 (42.9)	611 (37.6)	181 (37.8)	2556 (44.1)	1421 (39.5)	211 (34.6)
Former-smoker, N (%)	2343 (34.2)	1008 (36.4)	109 (27.4)	2780 (35.2)	533 (32.8)	147 (30.7)	2014 (34.7)	1245 (34.6)	201 (33.0)
Current smoker, N (%)	1590 (23.2)	676 (24.4)	98 (24.6)	1732 (21.9)	481 (29.6)	151 (31.5)	1231 (21.2)	936 (26.0)	197 (32.3)
**Education**									
Low, N (%)	895 (13.0)	591 (21.2)	129 (32.2)	767 (9.6)	633 (38.9)	215 (44.7)	469 (8.0)	875 (24.1)	271 (44.4)
Medium, N (%)	3528 (51.3)	1666 (59.8)	226 (56.4)	4338 (54.5)	849 (52.1)	233 (48.4)	2899 (49.7)	2212 (61.0)	309 (50.6)
High, N (%)	2444 (35.5)	527 (18.9)	46 (11.5)	2839 (35.7)	145 (8.9)	33 (6.9)	2449 (42.0)	537 (14.8)	31 (5.1)
Unclassifiable, N (%)	16 (.2)	3 (.1)		17 (.2)	2 (.1)		16 (.3)	3 (.1)	
**Monthly Income**									
Low income, N (%)	535 (7.8)	422 (15.2)	68 (16.9)	707 (8.9)	232 (14.3)	86 (17.9)	409 (7.0)	545 (15.1)	71 (11.7)
Medium income, N (%)	1900 (27.7)	909 (32.7)	116 (28.8)	2122 (26.7)	628 (38.6)	175 (36.4)	1457 (25.1)	1209 (33.4)	259 (42.5)
High income, N (%)	3600 (52.5)	1031 (37.1)	89 (22.1)	4173 (52.6)	438 (26.9)	109 (22.7)	3313 (57.0)	1241 (34.3)	166 (27.3)
Don’t know/Don’t tell, N (%)	825 (12.0)	418 (15.0)	130 (32.3)	934 (11.8)	328 (20.2)	111 (23.1)	636 (10.9)	624 (17.2)	113 (18.6)

S6 Table: Distribution of the population characteristics according to the different levels of occupational exposures (pesticides, solvents, and metals).

Population characteristics	Pesticides			Solvents			Metals		
	No	Low	High	No	Low	High	No	Low	High
**Age, median (min-max)**	46 (18-77)	46 (22-73)	45 (23-70)	46 (18-77)	46 (20-73)	44 (20-73)	46 (18-77)	44(22-66)	45 (20-70)
**BMI, median (min-max)**	26 (16-53)	26 (19-44)	26 (20-35)	26 (17-53)	26 (16-52)	27 (17-51)	25 (17-53)	26 (18-48)	26 (16-37)
**Sex**									
Male, N (%)	4172 (43.1)	237 (74.8)	78 (86.7)	3427 (45.7)	837 (37.2)	223 (64.5)	3807 (40.7)	457 (90.5)	223 (97.8)
Female, N(%)	5508 (56.9)	80 (25.2)	12 (13.3)	4065 (54.3)	1412 (62.8)	123 (35.5)	5547 (59.3)	48 (9.5)	5 (2.2)
**Smoking status**									
Never smoker, N (%)	3986 (41.5)	160 (51.0)	42 (46.7)	3128 (42.0)	941 (41.8)	119 (34.5)	3911 (42.1)	194 (38.6)	83 (36.6)
Former-smoker, N (%)	3352 (34.9)	83 (26.4)	25 (27.8)	2548 (34.3)	783 (34.8)	129 (37.4)	3233 (34.8)	149 (29.7)	78 (34.4)
Current smoker, N (%)	2270 (23.6)	71 (22.6)	23 (25.6)	1763 (23.7)	504 (22.4)	97 (28.1)	2139 (23.0)	159 (31.7)	66 (29.1)
**Education**									
Low, N (%)	1498 (15.5)	92 (29.1)	25 (28.1)	1198 (16.0)	303 (13.5)	114 (33.0)	1349 (14.4)	159 (31.5)	107(47.1)
Medium, N (%)	5188 (53.7)	175 (55.4)	57 (64.0)	3815 (51.0)	1383 (61.6)	222 (64.3)	5043 (54.0)	263 (52.1)	114 (50.2)
High, N (%)	2961 (30.6)	49 (15.5)	7 (7.9)	2451 (32.8)	557 (24.8)	9 (2.6)	2928 (31.4)	83 (16.4)	6 (2.6)
Unclassifiable, N (%)	19 (.2)			18 (.2)	1 (.0)		19 (.2)		
**Monthly Income**									
Low income, N (%)	951 (9.9)	58 (18.3)	16 (18.0)	706 (9.5)	276 (12.3)	43 (12.5)	945 (10.1)	54 (10.7)	26 (11.5)
Medium income, N (%)	2820 (29.3)	80 (25.2)	25 (28.1)	2134 (28.6)	662 (29.5)	129 (37.4)	2645 (28.4)	178 (35.3)	102 (44.9)
High income, N (%)	4632 (48.1)	70 (22.1)	18 (20.2)	3631 (48.7)	996 (44.4)	93 (27.0)	4477 (48.1)	186 (36.9)	57 (25.1)
Don’t know/Don’t tell, N (%)	1234 (12.8)	109 (34.4)	30 (33.7)	985 (13.2)	308 (13.7)	80 (23.2)	1245 (13.4)	86 (17.1)	42 (18.5)

S7 Table: Associations between occupational exposures and sickness absence.

Occupational exposures	Any SA[1]		Long-term SA[2]	
	OR (95% CI)	P-value	OR (95% CI)	P-value
**Biological dust**				
No exposure				
Low exposure	1.07 (0.97-1.18)	0.165	1.11 (0.95-1.28)	0.186
High exposure	**0.72 (0.58-0.89)**	**0.003**	0.90 (0.65-1.25)	0.533
**Mineral dust**				
No exposure				
Low exposure	**0.88 (0.78-0.99)**	**0.036**	0.98 (0.82-1.17)	0.814
High exposure	1.04 (0.85-1.28)	0.702	1.31 (0.98-1.76)	0.071
**Gasfumes**				
No exposure				
Low exposure	0.96 (0.89-1.05)	0.393	1.13 (0.98-1.30)	0.096
High exposure	1.07(0.89-1.28)	0.483	**1.46 (1.11-1.91)**	**0.006**
**Pesticides**				
No exposure				
Low exposure	**0.70 (0.55-0.89)**	**0.004**	0.82 (0.57-1.19)	0.305
High exposure	0.72 (0.46-1.13)	0.157	1.00 (0.53-1.89)	0.991
**Solvents**				
No exposure				
Low exposure	**1.14 (1.03-1.26)**	**0.009**	**1.26 (1.08-1.46)**	**0.003**
High exposure	1.13 (0.90-1.41)	0.296	1.02 (0.71-1.46)	0.924
**Metals**				
No exposure				
Low exposure	1.11 (0.92-1.35)	0.271	**1.44 (1.09-1.91)**	**0.011**
High exposure	**1.68 (1.26-2.24)**	**0.000**	**1.75 (1.15-2.67)**	**0.009**

No exposure as reference group.

[1] SA=Sickness absence; Association between occupational exposures and any sickness absence adjusted for age. sex. BMI. education. smoking status. and monthly income (n=9756).

[2] Association between occupational exposures and long-term sickness absence adjusted for age. sex. BMI. education. smoking status. and monthly income (n=5797).

OR=Odds Ratio; CI= Confidence Interval.

S8 Table: Association between airborne occupational exposures and sickness absence. In this model, all airborne exposures were adjusted to assess the effect of co-exposures.

**Occupational exposures**	**Any SA**		**Long-term SA**	
	**OR (95% CI)**	**P-value**	**OR (95% CI)**	**P-value**
**Biological dust**	1.14 (0.99-1.31)	0.069	1.05 (0.85-1.30)	0.625
**Mineral dust**	0.93 (0.80-1.09)	0.158	0.89 (0.70-1.12)	0.309
**Gases/fumes**	**0.86 (0.74-1.00)**	**0.046**	1.12 (0.89-1.39)	0.328
**Pesticides**	**0.71 (0.56-0.89)**	**0.004**	0.88 (0.62-1.25)	0.463
**Solvents**	1.11 (0.97-1.28)	0.124	1.04 (0.85-1.28)	0.694
**Metals**	**1.33 (1.08-1.64)**	**0.007**	**1.51 (1.11-2.07)**	**0.010**

S9 Table. Association between occupational exposures and respiratory symptoms. The logistic regression model was adjusted for age, sex, BMI, smoking, education, and monthly income.

**Occupational exposures**	**Chronic cough**		**Chronic phlegm**		**Dyspnea**	
	OR (95% CI)	P value	OR (95% CI)	P value	OR (95% CI)	P value
**Gases/fumes**						
No	Reference		Reference		Reference	
Low	1.09 (.96-1.24)	.170	1.04 (.90-1.21)	.567	1.01 (.88-1.17)	.842
High	1.18 (.94-1.50)	.159	1.29 (.99-1.67)	.055	1.21 (.91-1.62)	.191
**Solvents**						
No	Reference		Reference		Reference	
Low	.99 (.86-1.13)	.835	.95 (.81-1.12)	.539	.95 (.81-1.10)	.489
High	1.07 (.80-1.43)	.667	1.37 (1.00-1.88)	.049	1.05 (.74-1.50)	.771
**Metals**						
No	Reference		Reference		Reference	
Low	1.24 (.98-1.57)	.080	1.14 (.87-1.50)	.350	1.11 (.81-1.52)	.505
High	.88 (.60-1.27)	.488	1.18 (.80-1.75)	.406	1.46 (.95-2.24)	.086

OR=Odds Ratio; CI= Confidence Interval.

S10 Table: Association between respiratory symptoms and sickness absence. The logistic regression model was adjusted for age, sex, BMI, smoking, education, and monthly income.

**Respiratory symptoms**	**Any sickness absence**		**Long-term sickness absence**	
	OR (95% CI)	P-value	OR (95% CI)	P-value
**Chronic Cough**				
No	Reference		Reference	
Yes	**1.52 (1.35-1.71)**	**0.000**	**1.46 (1.22-1.73)**	**.000**
**Chronic Phlegm**				
No	Reference		Reference	
Yes	**1.59 (1.38-1.83)**	**0.000**	**1.74 (1.43-2.13)**	**.000**
**Dyspnea**				
No	Reference		Reference	
Yes	**1.46 (1.28-1.67)**	**0.000**	**1.70 (1.40-2.05)**	**.000**

OR=Odds Ratio; CI= Confidence Interval.

S11 Table: SNPs-by-Biological dust/Mineral dust/Gases and Fumes interactions on any sickness absence.

Variables in equation	Biological dust	Mineral dust			Gases and fumes		
	**rs17490056**<br>CC: n= 2449<br>(reference)<br>TC: n=4789<br>TT: n=2462	**rs13278529**<br>TT: n= 7105<br>(reference)<br>TG: n=2390<br>GG: n=205	**rs473892**<br>CC: n= 2907<br>(reference)<br>CT: n=4804<br>TT: n=1989	**rs6751439**<br>GG: n= 7351<br>(reference)<br>GA: n=2177<br>AA: n=172	**rs2888674**<br>GG: n= 2803<br>(reference)<br>GA: n=4822<br>AA: n=2075	**rs159497**<br>TT: n= 2763<br>(reference)<br>TC: n=4882<br>CC: n=2055	**rs516732**<br>TT: n= 2780<br>(reference)<br>TC: n=4818<br>CC: n=2102
	**OR (CI 95%)**	**OR (CI 95%)**	**OR (CI 95%)**	**OR (CI 95%)**	**OR (CI 95%)**	**OR (CI 95%)**	**OR (CI 95%)**
**Low exposure**	1.00 (0.83-1.20)<br>p = 0.980	**0.86 (0.75-0.99)<br>p = 0. 033**	**0.75 (0.61-0.93)<br>p = 0.008**	**0.87 (0.76-1.00)<br>p = 0.047**	1.17 (1.00-1.38)<br>p = 0.056	0.88 (0.74-1.03)<br>p = 0.116	0.93 (0.79-1.10)<br>p = 0.423
**High exposure**	**0.54 (0.36-0.83)<br>p = 0.005**	0.99 (0.78-1.26)<br>p = 0.934	0.94 (0.66-1.33)<br>p = 0.721	1.04 (0.83-1.32)<br>p = 0.707	1.40 (0.99-1.97)<br>p = 0.054	1.00 (0.72-1.38)<br>p = 0.994	0.93 (0.67-1.30)<br>p = 0.677
**SNP-HZ**	0.97 (0.86-1.09)<br>p = 0.574	0.94 (0.84-1.04)<br>p = 0.236	0.96 (0.86-1.06)<br>p = 0.402	1.03 (0.93-1.16)<br>p = 0.537	**1.14 (1.01-1.30)<br>p = 0.033**	0.95 (0.84-1.07)<br>p = 0.402	0.93 (0.82-1.05)<br>p = 0.251
**SNP-HM**	0.93 (0.81-1.07)<br>p = 0.313	1.13 (0.81-1.58)<br>p = 0.469	0.92 (0.89-1.05)<br>p = 0.214	0.91 (0.64-1.30)<br>p = 0.618	**1.29 (1.11-1.50)<br>p = 0.001**	0.84 (0.72-0.97)<br>p = 0.021	0.92 (0.79-1.07)<br>p = 0.278
**Low exposure by SNP-HZ**	1.13 (0.96-1.42)<br>p = 0.275	1.16 (0.89-1.50)<br>p = 0.271	1.16 (0.89-1.49)<br>p = 0.268	1.11 (0.85-1.45)<br>p = 0.429	0.82 (0.67-1.01)<br>p = 0.057	1.11 (0.91-1.36)<br>p = 0.295	1.02 (0.83-1.25)<br>p = 0.838
**Low exposure by SNP-HM**	1.05 (0.81-1.35)<br>p = 0.709	0.83 (0.41-1.65)<br>p = 0.593	**1.58 (1.15-2.18)<br>p = 0.005**	0.77 (0.34-1.76)<br>p = 0.543	**0.64 (0.50-0.81)<br>p = 0.000**	1.21 (0.95-1.55)<br>p = 0.127	1.11 (0.87-1.42)<br>p = 0.408
**High exposure by SNP-HZ**	1.47 (0.88-2.48)<br>p = 0.143	1.40 (0.90-2.17)<br>p = 0.131	1.12 (0.72-1.75)<br>p = 0.605	1.00 (0.63-1.60)<br>p = 0.983	0.67 (0.45-1.02)<br>p = 0.060	1.08 (0.72-1.61)<br>p = 0.710	1.23 (0.82-1.85)<br>p = 0.323
**High exposure by SNP-HM**	1.68 (0.91-3.07)<br>p = 0.094	0.26 (0.07-1.04)<br>p = 0.056	1.29 (0.75-2.21)<br>p = 0.353	1.19 (0.33-4.30)<br>p = 0.791	0.67 (0.40-1.12)<br>p = 0.130	1.03 (0.63-1.71)<br>p = 0.895	1.10 (0.68-1.78)<br>p = 0.689

Interactions analysis adjusted for age. sex. BMI. education. smoking status. and monthly income.
Biological dust (no exposure: n = 6650 (reference). low exposure: n = 2670. high exposure: n = 380)
Mineral dust (no exposure: n = 7679 (reference). low exposure: n = 1561. high exposure: n = 460)
Gases and fumes (no exposure: n = 5641 (reference). low exposure: n = 3469. high exposure: n = 590)
SNP-Single nucleotide polymorphism; HZ-Heterozygous; HM-Homozygous for minor allele. OR=Odds Ratio; CI= Confidence Interval.

S12 Table: SNPs-by-Biological dust/Mineral dust/Gases and Fumes interactions on long-term sickness absence.

Variables in equation	Biological dust	Mineral dust			Gases and fumes		
	**rs17490056** CC: n=1461 (reference) TC: n=2819 TT: n=1477	**rs13278529** TT: n=4206 (reference) TG: n=1432 GG: n=119	**rs473892** CC: n=1691 (reference) CT: n=2874 TT: n=1192	**rs6751439** GG: n=4343 (reference) GA: n=1297 AA: n=117	**rs2888674** GG: n=1704 (reference) GA: n=2850 AA: n=1203	**rs159497** TT: n=1615 (reference) TC: n=2894 CC: n=1248	**rs516732** TT: n=1629 (reference) TC: n=2889 CC: n=1239
	**OR (CI 95%)**	**OR (CI 95%)**	**OR (CI 95%)**	**OR (CI 95%)**	**OR (CI 95%)**	**OR (CI 95%)**	**OR (CI 95%)**
**Low exposure**	1.20 (0.91-1.58) p = 0.203	0.96 (0.78-1.19) p = 0.712	0.90 (0.65-1.24) p = 0.518	1.00 (0.81-1.23) p = 0.996	**1.29 (1.01-1.66) p = 0.044**	0.85 (0.65-1.11) p = 0.228	1.08 (0.84-1.40) p = 0.548
**High exposure**	0.95 (0.53-1.72) p = 0.870	1.27 (0.90-1.79) p = 0.175	1.14 (0.67-1.93) p = 0.636	1.22 (0.86-1.72) p = 0.266	1.79 (1.09-2.94) p = 0.021	1.28 (0.80-2.06) p = 0.301	1.23 (0.76-2.00) p = 0.399
**SNP-HZ**	1.04 (0.85-1.26) p = 0.731	0.95 (0.80-1.13) p = 0.536	1.07 (0.90-1.27) p = 0.469	1.14 (0.96-1.36) p = 0.136	1.03 (0.84-1.27) p = 0.753	0.88 (0.71-1.08) p = 0.215	0.94 (0.76-1.16) p = 0.572
**SNP-HM**	0.92 (0.73-1.15) p = 0.463	1.19 (0.70-2.02) p = 0.514	1.07 (0.87-1.33) p = 0.505	1.42 (0.86-2.36) p = 0.173	1.12 (0.87-1.44) p = 0.396	0.81 (0.63-1.04) p = 0.098	0.90 (0.69-1.16) p = 0.404
**Low exposure by SNP-HZ**	0.87 (0.62-1.22) p = 0.416	1.17 (0.80-1.73) p = 0.420	1.06 (0.71-1.57) p = 0.788	0.95 (0.63-1.41) p = 0.784	0.90 (0.66-1.23) p = 0.503	**1.51 (1.10-2.08) p = 0.012**	1.04 (0.76-1.43) p = 0.805
**Low exposure by SNP-HM**	0.94 (0.64-1.40) p = 0.773	0.23 (0.05-1.09) p = 0.064	1.32 (0.81-2.14) p = 0.266	0.48 (0.13-1.84) p = 0.286	**0.67 (0.45-0.98) p = 0.041**	1.39 (0.93-2.06) p = 0.105	1.11 (0.75-1.63) p = 0.610
**High exposure by SNP-HZ**	0.73 (0.34-1.60) p = 0.435	1.30 (0.69-2.43) p = 0.413	1.17 (0.61-2.24) p = 0.638	1.21 (0.64-2.28) p = 0.559	0.70 (0.39-1.28) p = 0.250	1.24 (0.70-2.21) p = 0.468	1.31 (0.72-2.37) p = 0.370
**High exposure by SNP-HM**	1.42 (0.61-3.32) p = 0.417	0.28 (0.03-2.39) p = 0.243	1.37 (0.63-2.99) p = 0.425	1.81 (0.41-8.00) p = 0.436	0.84 (0.41-1.73) p = 0.645	1.01 (0.48-2.15) p = 0.973	1.13 (0.56-2.29) p = 0.733

Interactions analysis adjusted for age. sex. BMI. education. smoking status. and monthly income.
Biological dust (no exposure: n = 3897 (reference). low exposure: n = 1592. high exposure: n = 268)
Mineral dust (no exposure: n = 4455 (reference). low exposure: n = 1008. high exposure: n = 294)
Gases and fumes (no exposure: n = 3226 (reference). low exposure: n = 2157. high exposure: n = 374)
SNP-Single nucleotide polymorphism; HZ-Heterozygous; HM-Homozygous for minor allele. OR=Odds Ratio; CI= Confidence Interval.

S13 Table: SNPs-by-Pesticides interactions on any sickness absence.

Variables in equation	**rs4764419** TT: n= 2786 (reference) TG: n=4886 GG: n=2028	**rs10459067** CC: n= 3056 (reference) CT: n=4836 TT: n=1808	**rs482555** TT: n= 5495 (reference) TC: n=3634 CC: n=571	**rs2145067** CC: n= 6676 (reference) . CT: n=2755. TT: n=269
	**OR (CI 95%)**	**OR (CI 95%)**	**OR (CI 95%)**	**OR (CI 95%)**
**Pesticides low exposure**	0.77 (0.51-1.18) p = 0.233	0.72 (0.48-1.08) p = 0.113	**0.68 (0.50-0.93) p = 0.016**	**0.69 (0.52-0.93) p = 0.014**
**Pesticides high exposure**	0.63 (0.25-1.55) p = 0.313	0.81 (0.37-1.78) p = 0.604	0.96 (0.52-1.79) p = 0.900	0.86 (0.50-1.48) p = 0.588
**SNP-HZ**	1.04 (0.95-1.15) p = 0.399	1.03 (0.94-1.13) p = 0.496	1.01 (0.93-1.11) p = 0.745	0.96 (0.87-1.05) p = 0.382
**SNP-HM**	1.01 (0.90-1.14) p = 0.854	1.01 (0.90-1.14) p = 0.863	1.03 (0.86-1.23) p = 0.775	1.11 (0.86-1.43) p = 0.429
**Pesticides low exposure by SNP-HZ**	0.86 (0.50-1.48) p = 0.586	1.0 (0.59-1.70) p = 0.993	1.12 (0.67-1.87) p = 0.657	1.15 (0.68-1.95) p = 0.611
**Pesticides low exposure by SNP-HM**	0.90 (0.45-1.80) p = 0.767	0.85 (0.41-1.75) p = 0.661	0.93 (0.33-2.66) p = 0.899	0.26 (0.03-2.32) p = 0.228
**Pesticides high exposure by SNP-HZ**	1.43 (0.47-4.31) p = 0.526	0.85 (0.31-2.39) p = 0.765	0.53 (0.21-1.34) p = 0.182	0.56 (0.19-1.60) p = 0.278
**Pesticides high exposure by SNP-HM**	0.85 (0.23-3.10) p = 0.800	0.80 (0.23-2.77) p = 0.722	0.83 (0.05-14.82) p = 0.902	0.50 (0.04-6.15) p = 0.590

Interactions analysis adjusted for age. sex. BMI. education. smoking status. and monthly income.
no exposure: n = 9312 (reference). low exposure: n = 304. high exposure: n = 84
SNP-Single nucleotide polymorphism; HZ-Heterozygous; HM-Homozygous for minor allele.
OR=Odds Ratio; CI= Confidence Interval.

S14 Table: SNPs-by-Pesticides interactions on long-term sickness absence.

Variables in equation	**rs4764419**<br>TT: n=1660<br>(reference)<br>TG: n=2899<br>GG: n=1198	**rs10459067**<br>CC: n=1827<br>(reference)<br>CT: n=2866<br>TT: n=1064	**rs482555**<br>TT: n=3281<br>(reference)<br>TC: n=2154<br>CC: n=322	**rs2145067**<br>CC: n=3979<br>(reference)<br>CT: n=1626<br>TT: n=152
	**OR (CI 95%)**	**OR (CI 95%)**	**OR (CI 95%)**	**OR (CI 95%)**
**Pesticides low exposure**	1.33 (0.76-2.33)<br>p = 0.319	1.13 (0.65-1.96)<br>p = 0.658	0.84 (0.53-1.33)<br>p = 0.456	0.78 (0.50-1.23)<br>p = 0.287
**Pesticides high exposure**	0.59 (0.13-2.70)<br>p = 0.493	0.75 (0.21-2.67)<br>p = 0.654	1.57 (0.68-3.62)<br>p = 0.288	1.45 (0.72-2.91)<br>p= 0.293
**SNP-HZ**	1.12 (0.96-1.31)<br>p = 0.149	1.07 (0.92-1.24)<br>p = 0.407	0.98 (0.8- 1.12)<br>p = 0.756	**0.86 (0.74-1.00)**<br>**p = 0.049**
**SNP-HM**	0.98 (0.81-1.19)<br>p = 0.844	0.94 (0.78-1.15)<br>p = 0.568	0.85 (0.63-1.15)<br>p = 0.297	0.94 (0.62-1.42)<br>p = 0.762
**Pesticides low exposure by SNP-HZ**	0.45 (0.20-1.00)<br>p = 0.049	0.60 (0.28-1.32)<br>p = 0.207	1.09 (0.50-2.38)<br>p = 0.826	1.30 (0.59-2.88)<br>p = 0.512
**Pesticides low exposure by SNP-HM**	0.48 (0.16-1.47)<br>p = 0.201	0.51 (0.15-1.68)<br>p = 0.265		
**Pesticides high exposure by SNP-HZ**	2.48 (0.44-13.95)<br>p = 0.302	1.69 (0.37-7.72)<br>p = 0.500	0.40 (0.11-1.46)<br>p = 0.165	
**Pesticides high exposure by SNP-HM**	1.03 (0.12-8.84)<br>p = 0.976	1.03 (0.14-7.60)<br>p = 0.976		1.02 (0.07-13.99)<br>p = 0.988

Interactions analysis adjusted for age. sex. BMI. education. smoking status. and monthly income.
no exposure: n = 5476 (reference). low exposure: n = 218. high exposure: n = 63
SNP-Single nucleotide polymorphism; HZ-Heterozygous; HM-Homozygous for minor allele.
No subjects in the blank cells; OR=Odds Ratio; CI= Confidence Interval.

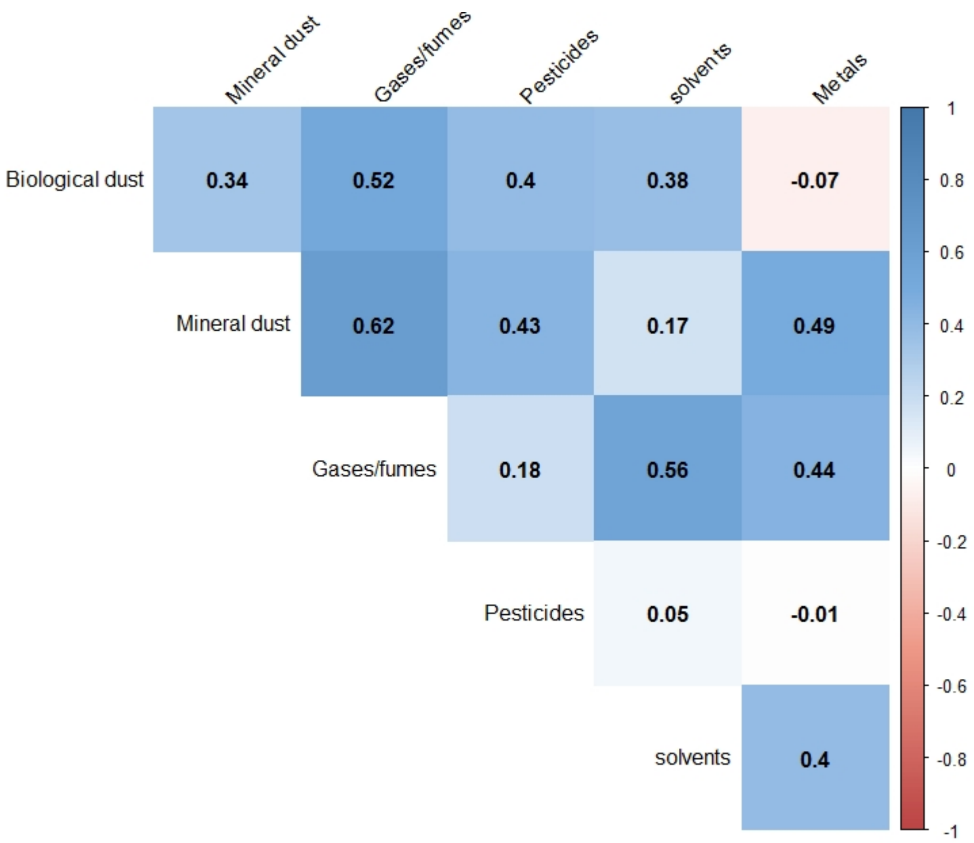

Figure S1: Correlogram shows the correlation (spearman rho) among airborne occupational exposures.
